# Association between Cullin-3 Single-Nucleotide Polymorphism rs17479770 and Essential Hypertension in the Male Chinese Han Population

**DOI:** 10.1155/2017/3062759

**Published:** 2017-07-18

**Authors:** Jin Li, Jing Hu, Rong Sun, Yongpan Zhao, Heping Liu, Jian Li, Lei Shi, Shujin Zhao

**Affiliations:** ^1^School of Bioscience and Bioengineering, South China University of Technology, Guangzhou, China; ^2^Department of Pharmacy, Guangzhou General Hospital of Guangzhou Military Command, Guangzhou, China; ^3^Department of Nephrology, Guangzhou General Hospital of Guangzhou Military Command, Guangzhou, China; ^4^Futian District Chronic Disease Prevention and Cure Center, Shenzhen, China

## Abstract

**Background:**

Hypertension, including essential and secondary hypertension, is a multifactorial disease, affecting more than one billion people worldwide. Secondary hypertension can result from mutations of cullin-3 (*CUL3*); however, whether polymorphisms of *CUL3* are associated with essential hypertension (EH) has not been reported. Here, we investigated the association between *CUL3* SNPs rs17479770 and rs3738952 and EH in the Chinese Han population.

**Methods:**

This case-control study investigated 520 representatives, including 259 patients with EH and 261 normotensive controls matched for age, gender, BMI, TG, TC, and HbA1c for the distribution of functional rs17479770 and rs3738952 within the *CUL3* gene by using PCR and RFLP.

**Results:**

Our results showed that there was no significant difference in allele and genotype distribution of rs3738952 and haplotype distribution of rs17479770 and rs3738952 between the EH group and normotensive group, whereas the rs17479770 TT genotype in male and the full data set were significantly associated with the decreased risk of EH (*P* = 0.050, *P* = 0.042), and rs17479770 allele T in male was shown to have the correlation tendency of the decreased risk of EH (*P* = 0.064).

**Conclusion:**

Our data suggest that the *CUL3* rs17479770 variant could be a protective factor in the pathogenesis of EH.

## 1. Introduction

Cullin-3 (CUL3) is the core component of multiple cullin-RING-based BCR (BTB-CUL3-RBX1) E3 ubiquitin-protein ligase complexes which mediate the ubiquitination and subsequent proteasomal degradation of target proteins [1]. The CUL3-RING E3 ubiquitin ligase (CRL) complex controls the ubiquitination of with-no-lysine kinase (WNK) and enhances the levels of WNK isoforms, whose function is similar to that of a serine-threonine protein kinase critical in controlling potassium, sodium, and pH homeostasis. The CRL complex also plays a major role in regulating blood pressure by increased activity of ion cotransporters in the kidney [2, 3]. Through stabilizing WNK isoforms, the mutation of *CUL3* has been linked to Pseudohypoaldosteronism type II (PHAII), a rare Mendelian syndrome featuring hypertension [4]. Vascular actions of CUL3 may contribute to hypertension, because McCormick and colleagues proposed that CUL3 regulates vascular tone via RhoA/Rho kinase signaling [5].

Hypertension is considered one of the most important diseases with a great burden on health care systems around the world. Hypertension is highly prevalent in the Asia-Pacific [6]. About 2 million 430 thousand people died of hypertension, accounting for about 24.6% of all deaths in China in 2010. In 2013, China's total health expenditure is 31869 billion yuan, of which the direct economic burden of hypertension accounted for 6.61% [7]. Essential hypertension (EH), which accounts for 90% of hypertensive cases, is a polygenic and multifactorial disease caused by the interaction of genetic determinants and environmental factors [8, 9]. The pathogenesis and etiology of EH include a multifactorial imbalance which results from complicated gene-gene and gene-environment interaction [10].

Single-nucleotide polymorphisms (SNPs) are the variation in the genomes which can be used to associate genotypic variation with the phenotype [11]. SNPs underlie differences in our susceptibility to disease. Associations of some SNPs in *WNK1* and *WNK4* with EH have been observed in the general population [12–14], and one study had identified that *rs3738952* of *CUL3* was significantly associated with head and neck squamous cell carcinoma (HNSCC) risk [15], but very few reports investigated the relationship between SNPs of *CUL3* and EH [16]. Genome linkage studies have identified numerous gene variants that associated with EH, and a few genetic loci and candidate genes (variants) have been identified by genome-wide association studies (GWAS) [17]. Through GWAS, a variety of common genetic variants are analyzed and identified for disease association, which have potential association with blood pressure and the development of EH [18, 19], whereas some gene variants have been shown to have contribution to EH according to ethnicity or gender [20]. The development of genetic studies has revealed that some SNPs within genes, such as *ATP1B1* [21], *CD36* [22], *CYP2J2* [23], *CYP4A11* [24, 25], *CYP4F2* [26], *CYP17A1* [27], and *TPRC6* [28], are closely related to the progression of EH.

However, it remains unknown whether there are other new gene variants which can influence the progression of EH. Therefore, we selected rs17479770 and rs3738952 SNPs in the *CUL3* gene after the haploview analysis in the Han population of China and examined the possible relationship between the SNPs and EH in this study. Haploview software was used to conduct linkage disequilibrium and haplotype block analyses, using the Hapmap phase IV genotype data for chromosomal region 2: 225043534–225157486 (CHB database, Hapmap release 24 (2008, November)). The criterion for r^2^ was set at 0.8. The Han population is the largest ethnic group in China, and the association of *CUL3* with EH in the Chinese Han population has not yet been reported. Therefore, our results could provide new insights into the pathogenesis of EH by studying *CUL3* SNPs.

## 2. Patients and Methods

### 2.1. Ethics

The present study was performed with the approval of the ethics committee of Guangzhou General Hospital of Guangzhou Military Command and is in compliance with the Helsinki Declaration. Informed consents were collected from all the candidate subjects.

### 2.2. Subjects

Patients diagnosed with EH were recruited from Guangzhou General Hospital of Guangzhou Military Command from 2012 to 2015. In total, 259 patients in the EH patient group and 261 control subjects in the normotensive group, matched for age, sex, BMI, TG, TC, and HbA1c, were enrolled in this study (Table 1). All of the EH patients received antihypertensive drug treatment, so it does not need to match the blood pressure between the two groups. All participants were unrelated and belonged to the Chinese Han population. EH patients were diagnosed according to JNC 7 [29] and JNC 8 [30]: systolic blood pressure (SBP) > 140 mmHg and diastolic blood pressure (DBP) > 90 mmHg, without any antihypertensive medication or confirmed diagnosis of EH by a cardiovascular specialist. The subjects who had secondary hypertension caused by another disease or are in the acute phase with cardiovascular, lung, liver, kidney, and other somatic diseases or with malignant tumor were excluded from the EH patient group. The normotensive group was selected based on the following criteria: SBP < 129 mmHg and DBP < 85 mmHg and without any antihypertensive medication. Subjects who had been currently diagnosed with malignant tumor and diabetes or in the acute phase with cardiovascular, lung, liver, kidney, and other somatic diseases were excluded from the normotensive group. One subject with diabetes in the EH patient group was excluded, and one subject with impaired glucose tolerance in the normotensive group was also excluded from analysis.

### 2.3. DNA Extraction

Genomic DNA was extracted from circulating leukocytes using commercial DNA isolation kits (Tiangen Biotech, Beijing, China). Briefly, the red blood cells, as well as the nuclei of leukocytes, were lysed. Subsequently, proteins were precipitated, followed by the precipitation of DNA using isopropanol. The DNA pellet was washed with ethanol. Finally, DNA was rehydrated with the DNA Rehydration Solution and preserved in liquid nitrogen.

### 2.4. Genotyping

Genomic DNA was isolated from peripheral blood leukocytes, according to the standard procedures by using commercial DNA isolation kits (Tiangen Biotech, Beijing, China). We performed genotyping for *CUL3* rs17479770 and rs3738952 polymorphisms by matrix-assisted laser desorption/ionization time-of-flight mass spectrometry (MALDI-TOF MS) by using the Sequenom Mass ARRAY system (Sequenom Inc., CA, USA). Primers used for genotyping were designed manually: rs17479770 forward 5′-TGCCA TTTCCTGCTAGCAACCT-3′ and reverse 5′-TCTTGGAAGGAAAGCTGTTGCATA-3′ and rs3738952 forward 5′-CCCAGGTCAACATAAATCACACATCA-3′ and reverse 5′-TTCTGCAGATCTCAATG CCACAT-3′. The concentration of rs17479770 primers was 1 *μ*M, and that of rs3738952 primers was 2 *μ*M in the PCR reaction system. PCR was performed in a reaction mixture volume of 20 *μ*L, which included 1× HotStarTaq buffer, 3.0 mM Mg^2+^, 0.3 mM dNTP, 1 U HotStarTaq polymerase (Qiagen Inc., MD, USA), and 1 *μ*L DNA template. Amplification was performed under the following conditions: initial denaturation of 2 min at 95°C followed by 11 cycles of denaturation at 94°C for 20 s, annealing at 59.5°C for 40 s, and extension at 72°C for 1 min and 30 s and 24 cycles of denaturation at 94°C for 20 s, annealing at 59°C for 30 s, and extension at 72°C for 1 min and 30 s followed by a final extension at 72°C for 2 min. PCR products were stored at 4°C. Restriction DNA fragments were separated by electrophoresis on 1% agarose gel and stained with ethidium bromide.

Linking primers used for coupled reaction for genotyping were designed manually: rs17479770RC, TTCCGCGTTCGGACTGATATTCAGCAAAATTAGAAGTCATTTCTAGTCCTGAG; rs17479770RP2, AGCAGAARTAATTAGAAATGTTAACATTTAAGTGCTTTTTTTTTTT; rs17479770RT, TACGGTTATTCGGGCTCCTGTTCAGCAAAATTAGAAGTCATTTCTAGTCCTGAA; rs3738952RC, TCTCTCGGGTCAATTCGTCCTTTCTCAATGCCACATTTTATGGACAAG; rs3738952RP, TTAAAAAGGTAAATATTGATAGTTTGAACGTATTAAGTAATTTTT; and rs3738952RT, TGTTCGTGGGCCGGATTAGTTCTCAATGCCACATTTTATGGACGAA. The reaction system of coupled reaction was performed in a reaction mixture system including 1 *μ*L 10× connection buffer, 0.25 *μ*L ligase, and 0.4 *μ*L 5′ primer mixture (1 connection M) and 0.4 *μ*L 3′ primer mixture (2 connection M), after purification of 2 *μ*L multiple PCR products and 6 *μ*L ddH_2_O mixture. Coupled reaction was performed under the following conditions: 38 cycles of denaturation at 94°C for 1 min and annealing at 56°C for 4 min. Reaction products were stored at 4°C. Allelic discrimination was measured automatically on the ABI3730XL (Applied Biosystems) using the GeneMapper 4.1 software (95% confidence intervals).

### 2.5. Statistical Analysis

Statistical analysis was performed using the SPSS 20.0 software (SPSS Inc., Chicago, IL, USA).

The haplotype of rs17479770 and rs3738952 was analyzed by PHASE 2.0 (University of Manchester, Manchester, UK). One-way ANOVA was used to match the values of BMI, HbA1c, TC, and TG between the EH patient group and normotensive group. The chi-squared test was used to examine whether the genotype distributions differed from the expected Hardy-Weinberg equilibrium (HWE) and the distribution of rs17479770 and rs3738952 genotypes and alleles between the EH patient group and normotensive group. Odds ratios (OR) and its corresponding 95% confidence intervals (CI) were estimated to compare the distribution of genotypes and alleles between the patients and control subjects. Analyses used two-tailed estimation of significance. *P* < 0.05 was considered to be statistically significant.

## 3. Results

### 3.1. Baseline Characteristics of the Study Population

The Han population consisted of 259 patients in the EH patient group (130 males and 129 females with an average age of 77.81 ± 7.640 years old) and 261 control subjects in the normotensive group (133 males and 128 females with an average age of 76.78 ± 9.095 years old), and there was no significant difference in age between the two groups (*P* = 0.163). The gender ratio of the two groups was insignificant (*P* = 0.930). The clinical representative characteristics including ID, age, gender, height, weight, TG, TC, and HbA1c were collected. There were no significant differences between the two groups including BMI, HbA1c, TC, and TG (*P* values were 0.297, 0.364, 0.111, and 0.324, resp.) (Table 1). The systolic blood pressure (SBP) and diastolic blood pressure (DBP) values were not included because all EH patients received antihypertensive medication.

### 3.2. Allele and Genotype Frequencies of *CUL3* rs17479770 and rs3738952 in the EH Patient Group and Normotensive Group

The allele and genotype frequencies of rs17479770 and rs3738952 SNPs are shown in Tables 2 and 3. Genotype distribution in the EH patient group and normotensive group did not deviate from HWE (*P* > 0.05). Comparison of allele frequencies between the patient group and normotensive group by the chi-squared test revealed that the allele and genotype frequencies of rs3738952 had no statistically significant difference between the EH patient group and the normotensive group (Table 3), whereas the rs17479770 TT genotype in males and the full data set had a significantly protective effects on EH (*P* = 0.050; OR = 0.578, 95% CI = 0.344–0.970; *P* = 0.042; OR = 0.674, 95% CI = 0.468–0.971) (Table 2) and the T allele in males and the CT genotype in the full data set show some protective trend of EH (*P* = 0.064; *P* = 0.066). When the data further stratified the haplotype frequency distributions of *CUL3* rs17479770 and rs3738952 SNPs, there is no statistically significant difference between the EH patient group and normotensive group (Table 4).

Overall, these results suggest that the TT genotype of rs17479770 in males and all representatives indicate a significantly protective effect on EH and the T allele shows some protective trend to the male representatives and the same as the CT genotype to all representatives.

## 4. Discussion

We investigated rs17479770 and rs3738952 SNPs in the *CUL3* gene as genetic risk factors for EH in a case-control study of a well-characterized Southern Chinese Han population. Our results demonstrate that the frequencies of the rs17479770 TT genotype in male EH patients and in all representatives were significantly decreased compared to those in the normotensive group, whereas there was no statistically significant difference in allele and genotype frequencies of *CUL3* in males between the EH patient group and the normotensive group. Moreover, there was no significant difference of haplotype frequency distributions of the two SNPs of *CUL3* in the EH patient group and normotensive group. The results of statistical analysis suggest that all *CUL3* rs17479770 TT genotypes were associated with the protection of the Chinese Han population especially male subjects from EH.

RhoA activation contributes to vascular constriction and hypertension, and CUL3Δ9-associated ubiquitin ligase activity toward RhoA is impaired, suggesting that CUL3Δ9 mutations may act dominantly by sequestering substrate adaptors and disrupting CUL3WT complexes [16]. CUL3 and KLHL3 are expressed in the distal nephron of the kidney, suggesting a mechanistic link between KLHL3 and CUL3 mutations, increased Na^+^-Cl^−^ reabsorption, and disease pathogenesis [31]. CUL3 provides a scaffold that binds to the BTB domain of *KLHL3* through its N-terminus region [32]. *WNK1* and *WKN4* regulate sodium and potassium flux through regulation of the thiazide-sensitive Na^+^/Cl^−^ cotransporter (NCC) and the renal outer medullary potassium channel (ROMK) in the distal nephron [33, 34]. The *WNK1* and *WNK4* isoforms, through directing two homologous kinases, SPS1-related proline/alanine-rich kinase (SPAK, also known as serine threonine kinase 39, STK39) and oxidative stress-responsive kinase1 (OSR1) which phosphorylates and activates NCC and Na^+^/K^+^/2Cl^−^ cotransporters (NKCC) 1 and 2, thereby play important roles in controlling blood pressure [35–38]. The kidney plays a central role in the pathophysiology of EH, and the NCC is physiologically relevant to the development of EH. Based on the previous studies, we inferred a mechanism that the mutations in *CUL3* might influence susceptibility to EH. In this study, we analyzed the association of *CUL3* rs17479770 and rs3738952 polymorphisms with EH in the Southern Chinese Han population, but several limitations of this study should be mentioned. The main limitation is the relatively small sample size: only 520 participants, including 259 EH patients and 261 normotensive people, were recruited, which is insufficient for an SNP association study of a rarer mutation site. The other limitation is that only two SNPs within the *CUL3* gene were analyzed. Additional in-depth studies are needed to confirm the functional importance of *CUL3* rs17479770 polymorphism in EH and to elucidate its precise role in the pathogenesis of EH.

## 5. Conclusion

This study demonstrates that *CUL3* rs17479770 is a candidate SNP that could be further examined as a possible protective genetic factor for EH progression, especially in male population. However, no significant association was detected between *CUL3* rs3738952 polymorphism and EH in the Chinese Han population, and the study of haplotype frequency distributions of *CUL3* rs17479770 and rs3738952 in the EH and normotensive groups had no significant association. Our results revealed that the *CUL3* rs17479770 TT genotype was associated with protection against EH in male and all representatives. This study offers a new direction to understand the mechanisms underlying EH and suggest novel therapeutic targets for the disease treatment. Further population-based genetic studies will be required to confirm our results and consolidate the role of CUL3 on EH in populations living in different environments or regions.

## Figures and Tables

**Table 1:** Baseline of the study population.

Parameters	EH group (*n* = 259)	Normotensive group (*n* = 261)	*P*
Age, y	77.81 ± 7.640	76.78 ± 9.095	0.163^∗^
Gender, male, %	50.2%	50.9%	0.930
BMI	23.654 ± 3.871	23.305 ± 3.754	0.297^∗^
HbA1c, %	5.3286 ± 0.901	5.258 ± 0.871	0.364^∗^
TC, mmol/L	4.448 ± 0.893	4.327 ± 0.836	0.111^∗^
TG, mmol/L	1.436 ± 0.926	1.346 ± 1.141	0.324^∗^

^∗^Analyzed by one-way ANOVA.

**Table 2:** Distribution frequency of *CUL3* rs17479770 polymorphism in the EH and control groups.

	*CUL3*	rs17479770	EH group	Normotensive group	*P*	OR (95% CI)
		(C/T)	*n*	*n*		
Full data set	Allele	C	229	207		
		T	289	317	0.132	1.213 (0.948–1.553)
	Genotype	CC	47	46	0.909	1.041 (0.665–1.630)
		CT	135	115	0.066	1.392 (0.986–1.965)
		TT	77	101	0.042	0.674 (0.468–0.971)
M	Allele	C	121	102		
		T	139	164	0.064	1.400 (0.989–1.980)
	Genotype	CC	27	22	0.430	1.323 (0.709–2.467)
		CT	67	58	0.218	1.375 (0.846–2.235)
		TT	36	53	0.050	0.578 (0.344–0.970)
F	Allele	C	108	107		
		T	150	151	1.000	1.016 (0.716–1.442)
	Genotype	CC	20	25	0.512	0.763 (0.400–1.457)
		CT	68	57	0.213	1.408 (0.863–2.299)
		TT	41	47	0.512	0.813 (0.485–1.361)

**Table 3:** Distribution frequency of *CUL3* rs3738952 polymorphism in the EH and control groups.

	*CUL3*	rs3738952	EH group	Normotensive group	*P*	OR (95% CI)
		(C/T)	*n*	*n*		
Full data set	Allele	C	387	399		
		T	131	123	0.564	0.911 (0.686–1.209)
	Genotype	CC	145	151	0.723	0.927 (0.655–1.311)
		CT	97	97	1.000	1.012 (0.710–1.444)
		TT	17	13	0.459	1.340 (0.637–2.819)
M	Allele	C	201	204	0.368	1.202 (0.810–1.786)
		T	59	72		
	Genotype	CC	76	79	0.901	0.962 (0.589–1.572)
		CT	49	46	0.610	1.144 (0.692–1.893)
		TT	5	8	0.572	0.625 (0.199–1.963)
F	Allele	C	186	195	0.315	0.808 (0.544–1.201)
		T	72	61		
	Genotype	CC	69	72	0.707	0.894 (0.547–1.462)
		CT	48	51	0.702	0.895 (0.541–1.479)
		TT	12	5	0.130	2.523 (0.862–7.382)

**Table 4:** Haplotype frequency distributions of the 2 SNPs of *CUL*3 in the EH and normotensive groups.

*CUL3*	Haplotype	EH group ratios	Normotensive group ratios	*χ* ^2^	*P* value	OR (95% CI)
Full data set	CC	229 : 259	207 : 261	0.700	0.436	1.115 (0.864–1.438)
	TC	158 : 259	192 : 261	1.824	0.189	0.829 (0.632–1.088)
	TT	131 : 259	123 : 261	0.213	0.647	1.073 (0.795–1.449)
Male	CC	121 : 130	102 : 133	1.128	0.317	1.214 (0.849–1.735)
	TC	80 : 130	102 : 133	1.293	0.288	0.802 (0.549–1.173)
	TT	59 : 130	62 : 133	0.015	0.913	0.974 (0.633–1.498)
Female	CC	108 : 129	105 : 128	0.012	0.926	1.021 (0.710–1.468)
	TC	78 : 129	90 : 128	0.577	0.488	0.860 (0.583–1.270)
	TT	72 : 129	61 : 128	0.545	0.521	1.171 (0.770–1.782)

## Abbreviations

ANOVA: Analysis of variance
BMI: Body mass index
BP: Blood pressure
BTB: Bric-a-brac tramtrack broad complex
CI: Confidence intervals
CRL: Cullin-RING E3 ubiquitin ligase
CUL3: Cullin-3
CYP: Cytochrome P450
DBP: Diastolic blood pressure
EH: Essential hypertension
GWAS: Genome-wide association studies
HbA1c: Hemoglobin A1c
HNSCC: Head and neck squamous cell carcinoma
HWE: Hardy-Weinberg equilibrium
JNC 7: Seventh report of the Joint National Committee on prevention, election, evaluation, and treatment of high blood pressure
JNC 8: 2014 evidence-based guideline for the management of high blood pressure in adults: report from the panel members appointed to the Eighth Joint National Committee
KLHL3: Kelch-like 3
MALDI-TOF MS: Matrix-assisted laser desorption/ionization time-of-flight mass spectrometry
NCC: Na^+^/Cl^−^ cotransporter
NKCC: Na^+^/K^+^/2Cl^−^ cotransporter
OR: Odds ratios
PCR: Polymerase chain reaction
PHAII: Pseudohypoaldosteronism type II
RFLP: Restriction fragment length polymorphism
ROMK: Renal outer medullary potassium channel
SBP: Systolic blood pressure
SNPs: Single-nucleotide polymorphisms
TC: Total cholesterol
TG: Triglyceride
WNK: With-no-lysine kinase.

## Additional Points

*Highlights.* The *CUL3* rs17479770 TT genotype was associated with protection from EH in male and in the full data set. *CUL3* rs17479770 is a possible protective genetic factor for EH progression.
